# The Interaction Effect between Blood Stasis Constitution and Atherosclerotic Factors on Cognitive Impairment in Elderly People

**DOI:** 10.1155/2018/8914090

**Published:** 2018-11-11

**Authors:** Zhizhen Liu, Hongqing Yang, Mozhu Zhang, Jing Cai, Zijie Huang

**Affiliations:** ^1^College of Integrative Medicine, Fujian University of Traditional Chinese Medicine, Fuzhou 350122, China; ^2^Community Health Service Center, Luoxing Street, Mawei District, Fuzhou 350015, China

## Abstract

**Objective:**

Blood stasis (BS) constitution represents a tendency to stagnation and positively associates with the severity of atherosclerosis. In this study, we have identified the interaction effect between BS constitution and atherosclerosis on cognitive impairment in the elderly people.

**Methods:**

Eligible elderly people ≥65 years old who attended physical examination in the Mawei community of Fuzhou city during 2015 were enrolled in this study. We explored the characteristics of Traditional Chinese Medicine (TCM) constitution and atherosclerotic factors in the normal and cognitive impairment groups and their interaction effect between participants' Minimental State Examination (MMSE) scores in the elderly people.

**Results:**

The prevalence of cognitive impairment in the elderly people was 13.0%. Red blood cell (RBC), hemoglobin (HB), ankle brachial index (ABI), brachial-ankle pulse wave velocity (BaPWV), and blood stasis (BS) were significantly different between normal and cognitive impairment group (P<0.05). Logistic regression analysis indicated that RBC (odds ratio (OR)=0.530 (0.343-0.817), P=0.004), HB (OR=0.980 (0.967-0.993), P=0.003), ABI (OR=2.199(1.112-4.347), P=0.023), and blood stasis constitution (OR=1.808 (1.022-3.202), P=0.042) were correlated with cognitive impairment. The interactions of blood stasis with HB, ABI, and BaPWV significantly impacted the MMSE score (P<0.05).

**Conclusion:**

Elderly individuals with blood stasis may be at a higher risk of arterial stenosis and sclerosis, leading to susceptibility to cognitive impairment.

## 1. Introduction

Cognitive impairment generally refers to various degrees of compromised cognitive function due to diverse factors, ranging from mild cognitive impairment to dementia. Along with global aging, the incidence of cognitive impairment is increasing [1]. At present, one of the major challenges in public health is to identify the risk of cognitive impairment in elderly populations and to take proper preventive measures, for maximizing benefits and minimizing the risk for the elderly population. So far, a number of studies have suggested that atherosclerotic factors, including age, sex, hypertension, hyperlipidemia, diabetes, ankle brachial index (ABI), brachial-ankle pulse wave velocity (BaPWV), smoking, obesity, and lack of exercise, are closely associated with cognitive impairment and increases the odds of the disease [2, 3].

During human life, Traditional Chinese Medicine (TCM) constitution is a comprehensive and relative stable trait based on the congenital and acquired morphological structure, physiological function, and psychological status [4]. The differences in individual TCM constitution determine the differential susceptibility and tendency to certain diseases [4], leading to different onsets of cognitive impairment. Thus, delaying the onset or progression should follow the principle of suiting measures according to different TCM constitutions [4].

Blood stasis (BS) constitution is a common TCM constitution in the elderly population with cognitive impairment [5, 6]. It represents a tendency to the stagnation of blood and may cause atherosclerosis [7]. Intuitively, BS constitution is typically considered to correlate with cardiovascular events; however the association between BS constitution and atherosclerosis caused by cognitive impairment remains unclear. In the current study, we used a cross-sectional design to investigate the possible interaction effect between BS constitution and atherosclerosis on cognitive function.

## 2. Methods

### 2.1. Subjects

**Figure 1:**
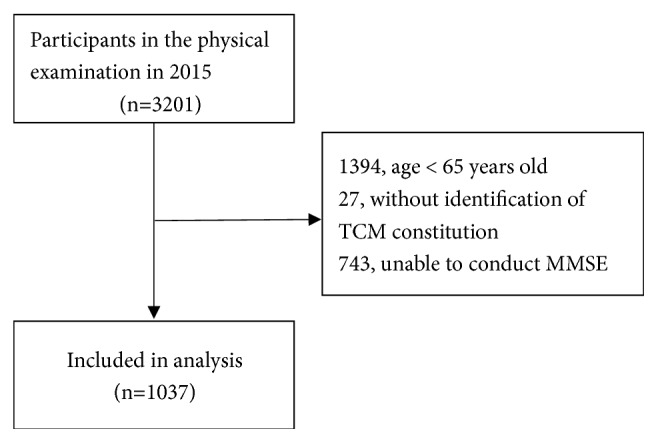
Flowchart of data processing and screening.

Inclusion criteria include patients *⩾*65 years old; residence for more than one year; attended physical examination organized by the Luoxing Street Community Health Service Center in 2015. Exclusion criteria include temporary residence in Luoxing Street, Mawei District (including 10 communities and villages: Yanshan Community, Maxian Community, Peiying Community, Xingang Community, Luoxing Community, Junzhu Village, Shangqi Village, Luojian Village, Qingzhou Village, and Luoxing Village); presence of audio-visual impairment; unable to perform identification of TCM constitution or MMSE; incomplete physical examination results. Written informed consent was obtained from each participant. This retrospective observational study (No. 2017-024) was approved by the institutional review board of the Fujian University of Traditional Chinese Medicine (Fujian, China). A total of 1,037 out of 3,201 subjects were included in this study (Figure 1).

### 2.2. Investigation Items

#### 2.2.1. General Information

Sociodemographic data are sex, age, education level, smoking (in the past 12 months), alcohol consumption (none, occasionally, often, or alcoholic), and exercise (none, occasionally, or often).

History of chronic illnesses: the diagnostic criteria of hypertension referred to China's Guidelines for Prevention and Control of Hypertension (2010 edition) [8]: systolic pressure *⩾*140 mmHg and/or diastolic pressure *⩾*90 mmHg, or with confirmed history of hypertension, or with normal blood pressure after oral antihypertensive medication. Diagnostic criteria of diabetes [9] are presence of typical diabetic symptoms (polyuria, polydipsia, and polyphagia) and random blood glucose level (anytime during a day) *⩾*11.1 mmol/L or fasting blood glucose (free of calorie intake for over 8 h) *⩾*7.0 mmol/L or blood glucose level 2 h after a glucose load *⩾*11.1 mmol/L.

#### 2.2.2. Atherosclerotic Factors

Red blood cells (RBC) and hemoglobin (HB) [10]: blood samples were examined by a Mindray BC5300 five-category hematology analyzer and reagents.

Blood lipids and glucose: blood lipid and glucose levels were examined by a Mindray BS420 automatic biochemical analyzer (Shenzhen, China). Related reagents were purchased from Purebio Biotechnology Co., Ltd. (Ningbo, China). Fasting venous blood (5 ml) was drawn from all subjects. Total cholesterol and triglyceride levels were measured by an enzyme-coupled assay. High-density lipoprotein (HDL) and low-density lipoprotein (LDL) were measured by an enzyme-multiplied immune assay.

ABI and BaPWV: limb bilateral ABI and BaPWV were determined using an AS-1000 PWV Measurement System (Heath Digit, Hong Kong, China). The reference value of BaPWV was set at 14.00 m/s; a higher value indicated a stiffer arterial wall. Diagnostic criteria of lower extremity arteriosclerosis include arteriosclerosis, ABI*⩾*1.3; normal, 1.0⩽ABI<1.3; critical range, 0.9<ABI<1.0; arteriostenosis, ABI⩽0.9. Examination procedures: the subject lays on the bed; the two upper limb cuffs were wrapped around the upper arms (air tubes going downwards to the hands), and the tow lower limb cuffs were wrapped around the shins (next to the ankles, air tubes going upwards along the fibula) for the measurement of blood pressure; BaPWV was measured with the cuffs stayed in the same positions.

#### 2.2.3. MMSE

MMSE, created by Folstein* et al.* in 1975[11], is the most common tool for the screening of cognitive impairment. This study adopted the Chinese version edited by M.Y. Zhang [12]. The MMSE included 19 major items (see Appendix 1), including orientation, memory, computation, attention, memory, and language, which were quantified as MMSE scores ranging from 0 to 30. Cognitive impairment was defined as ⩽17 for the illiteracy group, ⩽20 for the primary education group, and ⩽24 for the groups of secondary education and above. Subjects were therefore classified into a cognitive impairment group and a normal cognitive function group.

#### 2.2.4. Identification of TCM Constitution

According to the 33-item questionnaire in the TCM Service Log for the Elderly issued by China TCM Administration in 2013 [13] (see Appendix 2), general information was collected, and the TCM constitution was identified. The types of TCM constitution included unbalanced constitution (Qi-deficiency, Yang-deficiency, Yin-deficiency, phlegm, damp-heat, blood stasis, Qi-stagnation, and Inherited Special constitution) and balanced constitution. Criteria for the identification of biased constitution were as follows: “Yes” if the cumulative score of all items *⩾*11; “Tendency” if the cumulative score of all items =9-10; “No” if the cumulative score of all items ⩽ 8. Moreover, criteria for the identification of gentle constitution were as follows: “Yes” if the cumulative score of all items *⩾*17 and the final score of each of the other eight constitutions < 8; “Roughly yes” if the cumulative score of all items *⩾*17 and the final score of each of the other eight constitutions <10. Identification of TCM constitution was performed by trained TCM physicians.

### 2.3. Statistical Analyses

Statistical analysis of the data was performed using SPSS 24.0 software (SPSS Inc., Chicago, IL, USA). Quantitative data with a normal distribution were presented as the mean ± standard deviation, whereas quantitative data that did not meet a normal distribution were presented as the median and interquartile range. Enumeration data were presented as absolute number and rate. Group-wise comparisons of quantitative data with normal distribution were conducted by t-test. Quantitative data that did not meet a normal distribution were compared using Rank-sum test. Group-wise comparisons of enumeration data were performed using the Chi-square test. Ranked data were compared with the Rank-sum test. For categorical variables, the group with the lowest risk was used as the reference, and the odds ratio (OR) and 95% confidence interval (CI) of each stratum were obtained by setting dummy variables in a binary logistic regression model. The interaction effects of sociodemographic and atherosclerotic factors on MMSE score were analyzed using multivariate linear regression [14]. P<0.05 was considered significant in two-tail tests.

## 3. Results

### 3.1. Cognitive Impairment Conditions

After sending an invitation letter to older adults, n=3,201 individuals registered for the physical examination. Subjects who were < 65 years old (n=1,394) or who had incomplete data for diagnostic purposes (n=27+743) were excluded from the study. Finally, a total of 1,037 eligible subjects were included into the analysis. The 1,037 subjects scored 0-30 in MMSE and the average MMSE score was 25.06±4.42. A total of 135 subjects (13.0%) were characterized with cognitive impairment.

### 3.2. Cognitive Impairment Analysis and General Information

As shown in Table 1, the rate of cognitive impairment was compared according to sex, age, smoking history, exercise habit, and education level. The rates were significantly different on all these factors (P<0.05). Regarding medical history, the rate of cognitive impairment was not significantly different between the presence and absence of hypertension or diabetes (P>0.05).

### 3.3. Cognitive Impairment Analysis and Atherosclerotic Factors

Compared with individuals in the group with normal cognitive function, individuals in the cognitive impairment group had a higher BaPWV level (P < 0.05) and reduced levels of RBC, HB, and ABI (P < 0.05) (Table 2)

### 3.4. Cognitive Impairment and TCM Constitutions

Univariate analyses showed that the BS constitution variable was associated with cognitive function significantly (P<0.05, Table 3(a)). The subgroups of BS constitution scored differently in MMSE (P<0.05). MMSE score decreased with an increasing degree of BS (Table 3(b)).

### 3.5. Regression Analysis of BS Constitution and Levels of RBC, HB, ABI, and BaPWV

After controlling for sociodemographic factors, such as sex, age, smoking, education level, and exercise habit, logistic regression analysis indicated that RBC (OR=0.503, P=0.004), HB (OR=0.980, P=0.003), ABI ⩽0.90 (OR=2.199, P=0.023), and BS constitution (OR=1.808, P=0.042) correlated with cognitive impairment (Table 4).

### 3.6. Interaction Effect between BS Constitution and Atherosclerotic Factors

Hierarchical regression was employed to study the interaction effect between BS constitution and atherosclerotic factors on MMSE score. In order to avoid the influence of collinearity, variables were decentralized to obtain the corresponding standard scores. Subsequently, BS constitution was multiplied by standard scores of the atherosclerotic factors that displayed statistical significance in Table 2 in order to obtain the values of the interactive items “BS × atherosclerotic factors”. The values were then subjected to stratified regression. As shown in Table 5, the regression coefficients of the interactive items “BS × HB”, “blood stasis × ABI”, and “blood stasis × BaPWV” were statistically significant (P<0.05). Diagnosis of collinearity indicated that no significant collinearity existed among the studied factors.

**Figure 2:**
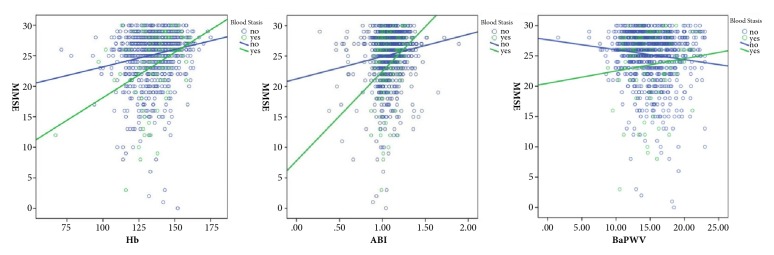
Interaction effect between blood stasis and atherosclerotic factors on MMSE score.

In order to better present the interactions with blood stasis constitution, all subjects were assigned to two groups: BS constitution group and non-BS group (Normal + Tendency). The fitting curves of regression equations for atherosclerotic factors and MMSE score were plotted for each group. As shown in Figure 2, the MMSE score in HB, ABI, and BaPWV level decreased even further in the presence of concurrent BS constitution.

## 4. Discussion

Different TCM constitutions may be encountered in patients diagnosed according to Western practices as having a similar disease. In the present study, a total of 135 subjects (13.0%) were characterized with cognitive impairment, and 17 of those (12.6%) involved BS constitution. This was consistent with the TCM etiology of cognitive impairment originates from deficiency in viscera and presents as blood stasis and phlegm obstruction [15, 16]. Moreover, BS is one of the key regulatory factors in the development of cognitive impairment. Stable BS, if not intervened promptly, may block the flow of Qi and blood through main and collateral channels in a long run and exacerbate Yin-Yang-deficiency, resulting in blockade of cerebral vessels, failure of YANG-orifice nourishing, memory descent, and accelerated progression of cognitive impairment.

Individuals with BS constitution tend to express blood high viscosity syndrome (BHVS), which is the pathological state of blood stagnation and associated with increased atherosclerosis [17]. In previous studies, it was found that ABI and BaPWV were reliable quantitative indicators of atherosclerosis [18, 19]. Reduced ABI and elevated BaPWV have been shown to increase the risk of cardiovascular events [19, 20]. In addition, reduced levels of HB and RBC closely associated with atherosclerosis and cognitive impairment in the elderly [21]. However, these studies did not distinguish between the interaction effect between BS constitution and atherosclerotic factors. We adjusted for sociodemographic factors using multivariate logistic regression analysis, and our results indicated that BS constitution was independently associated with atherosclerosis in patients with cognitive impairment. Results of the present study indicated that BS constitution promotes the formation of atherosclerosis through mechanisms that are different from that of traditional cardiovascular risk factors [22, 23].

The interaction between BS constitution and atherosclerotic factors was further investigated. The results showed that there were interactions of BS with HB, RBC, and ABI on cognitive impairment (P<0.05). These findings demonstrated that the MMSE score in HB, ABI, and BaPWV level decreased even further in the presence of concurrent BS constitution. The possible reason is that a relatively stable constitution has formed in adults and blood stasis exists throughout the whole process of disease development. Thus, blood stasis is a product of disease development, and it in turn causes further damage in the target organ(s). On the other hand, the nature of hemorheology changes with age [24]. These changes may cause high viscosity and hypercoagulability as well as high risks of arteriostenosis and atherosclerosis in the elderly population with BS, leading to susceptibility and tendency to cognitive impairment despite the progression of chronic diseases.

Serum lipid and glucose levels have frequently been reported as risk factors for cognitive impairment [25, 26]. However, in this study, TC, triglyceride, HDL-C, LDL-C, and glucose did not significantly differ between subjects with or without cognitive impairment. This may be due because the subjects involved a population with chronic diseases and long-term medications in these communities, and medications may interfere with the physical examination data.

*Limitations. *Based on cross-sectional data, in this study, we investigated the effect of TCM constitution-conditioned atherosclerotic factors on cognitive impairment in the elderly population. Although the cross-sectional data provided characteristics of TCM constitution of cognitive impairment in the elderly, the effect of early factors on TCM constitution is missed, and the current data fail to evaluate the reliability of all influencing factors. Therefore, a longitudinal study will be conducted based on the communities in order to obtain panel data for comprehensive understanding of the factors that influence TCM constitution in the elderly population with cognitive impairment. In addition, we only focused on single main TCM constitution of cognitive impairment in the elderly. Several participants had combined unbalanced constitutions with two or more types. The combined and interaction effects of TCM constitution types were not considered in this study. Therefore, this may confound the results. Furthermore, it is worth noting that the MMSE is poor at identifying mild cognitive impairment and less sensitive to cognitive decline as a follow-up tool [27]. Therefore, additional specific cognitive function testing tools will be used in our future studies.

## 5. Conclusion

In this study, we have shown that the elderly population with BS constitution have a higher risk of arterial stenosis and sclerosis, leading to susceptibility to cognitive impairment. Hence, it is important that community health service institutes shall not only provide basic medical treatment for chronic diseases in the elderly with a high risk of cognitive impairment, but also offer TCM intervention to promote blood circulation and remove BS according to the TCM constitution under the TCM principle of preventive treatment, for the best control of risk factors of cognitive impairment.

## Figures and Tables

**Table 1:** Distribution of general factors in aged subjects with and without cognitive impairment (n=1,037).

Item		Cognitive impairment		*χ* ^2^ value	P-value
		Normal	Presence		
**Sex**	Male	502 (90.0)	56 (10.0)	9.490	0.002
	Female	400 (83.5)	79 (16.5)		
**Age**	<70	491 (89.9)	55 (10.1)	8.833	0.003
	≥70	411 (83.7)	80 (16.3)		
**Smoking**	No	777 (86.1)	125 (13.9)	4.498	0.034
	Yes	109 (93.2)	8 (6.80)		
**Alcohol consumption**	None	786 (86.2)	126 (13.8)	2.720 Tendency	0.099
	Occasionally	72 (94.7)	4 (5.30)		
	Often	44 (89.8)	5 (10.2)		
**Education level**	Illiteracy	118 (65.2)	63 (34.8)	45.863 Tendency	<0.001
	Primary	318 (93.0)	24 (7.0)		
	Secondary	286 (88.5)	37 (11.5)		
	High school and above	180 (94.2)	11 (5.8)		
**Exercise**	Often	38 (82.6)	8 (17.4)	11.755	0.008
	Everyday	677 (88.7)	86 (11.3)		
	Occasionally	110 (85.9)	18 (14.1)		
	None	77 (77.0)	23 (23.0)		
**BMI**	Normal	446 (87.8)	62 (12.2)	2.551 Tendency	0.110
	Overweight	361 (87.8)	50 (12.2)		
	Obesity	95 (80.5)	23 (19.5)		
**Hypertension**	Absence	362 (88.3)	48 (11.7)	1.029	0.310
	Presence	540 (86.1)	87 (13.9)		
**Diabetes**	Absence	683 (87.5)	98 (12.5)	0.618	0.432
	Presence	219 (85.5)	37 (14.5)		

Note: the data are presented as number of subjects (rate, %).

**Table 2:** Distribution of atherosclerotic factors in the elderly population with or without cognitive impairment (n=1037).

Item (reference range)	Cognitive impairment		Z value	P-value
	Normal	Presence		
**RBC**(3.8-5.1 10*∗*12/L)	4.30 (0.54)	4.18 (0.54)	-2.518	**0.012**
**HB**(115-150 g/L)	136.0 (23.0)	132.5 (16.0)	-2.802	**0.005**
FBS (3.89-6.11mmol/L)	5.47 (1.21)	5.53 (1.53)	-0.451	0.652
TC (0-5.2 mmol/L)	5.09 (1.31)	5.09 (1.67)	-0.387	0.699
TG (0-1.7 mmol/L)	1.40 (0.90)	1.36 (0.81)	-0.333	0.739
LDL-C (0-3.12 mmol/L)	2.83 (1.01)	2.89 (1.06)	-0.083	0.934
HDL-C (0.91-2.59 mmol/L)	1.42 (0.43)	1.43 (0.44)	-0.266	0.790
**BaPWV** (10.6–21.4 m/s)	14.64 (3.98)	15.36 (3.87)	-2.077	**0.038**
**ABI **(1-1.3 m/s)	1.08 (0.17)	1.02 (0.14)	-3.933	**<0.001**

Note: the data are presented as median (interquartile range).

**Table:** (a) Distribution of cognitive impairment in various TCM constitutions of aged population (n=1037)

TCM constitution		Cognitive impairment		*χ* ^2^ value	P-value
		Normal	Presence		
Balanced	No	727(86.2)	116(13.8)	2.191	0.139
	Yes	175(90.2)	19(9.8)		
Qi-deficiency	No	821 (87.3)	119 (12.7)	1.142	0.285
	Yes	81 (83.5)	16 (16.5)		
Yang-deficiency	No	738 (87.9)	102 (12.1)	2.993	0.084
	Yes	164 (83.2)	33 (16.8)		
Yin-deficiency	No	656 (88.1)	89 (11.9)	2.685	0.101
	Yes	246 (84.2)	46 (15.8)		
Phlegm	No	627 (87.2)	92 (12.8)	0.103	0.749
	Yes	275 (86.5)	43 (13.5)		
Damp-heat	No	865 (86.9)	130 (13.1)	0.048	0.827
	Yes	37 (88.1)	5 (11.9)		
**Blood-stasis**	No	835 (87.6)	118 (12.4)	4.208	0.040
	Yes	67 (79.8)	17 (20.2)		
Qi-stagnation	No	872 (87.4)	126 (12.6)	3.621	0.057
	Yes	39(76.9)	9(23.1)		
Inherited Special	No	897(87.1)	133(12.9)		0.228
	Yes	5 (71.4)	2 (13.0)		

**Table:** (b) Distribution of MMSE scores in various TCM constitutions of aged population with cognitive impairment (n=135)

TCM constitution	MMSE score			*χ* ^2^ value	P-value
	Normal	Tendency	Presence		
Balanced	16.61±5.74	17.77±5.32	18.74±4.19	3.076	0.215
Qi-deficiency	17.50±5.06	18.11±4.32	14.13±7.99	2.222	0.329
Yang-deficiency	17.16±5.37	18.10±5.76	16.94±5.79	0.407	0.816
Yin-deficiency	17.40±5.60	17.62±6.11	16.59±4.86	2.047	0.359
Phlegm	17.23±4.63	16.44±7.06	17.65±5.29	0.208	0.901
Damp-heat	17.14±5.45	17.50±6.14	17.20±5.17	0.254	0.881
**Blood-stasis**	**17.72±5.35**	**16.81±5.23**	**14.29±5.78**	**6.853**	**0.032**
Qi-stagnation	17.53±5.02	13.83±9.09	14.78±7.61	1.567	0.457
Inherited Special	17.24±5.38	13.67±10.5	18.50±3.54	0.428	0.807

Note: as some subjects presented several TCM constitutions, i.e., they met the criteria of two or more TCM constitutions, they were included in the table as two TCM constitutions. For example, if a subject was identified as both Qi-deficiency constitution and phlegm constitution, the patient was included in the Qi-deficiency group as well as the phlegm group. If the subject did not meet the criteria of “Yes” for that constitution type, they were not included in the statistical analysis.

**Table 4:** Logistic regression analysis of cognitive impairment as a function of atherosclerotic factors and blood-stasis.

Items	OR (95% CI)^a^	P-value
RBC	0.530 (0.343-0.817)	0.004
HB	0.980 (0.967-0.993)	0.003
BaPWV		
<14 Normal	1.00	
≥14 Atherosclerosis	1.441 (0.982-2.115)	0.062
ABI		
1.0~1.3 Normal	1.00	
0.9~1.0 Critical	1.747 (1.160-2.629)	0.008
≤0.9 Arteriostenosis	2.199 (1.112-4.347)	0.023
≥1.3 Atherosclerosis	1.132 (0.495-2.587)	0.769
Blood stasis		
Absence	1.00	
Tendency	1.056 (0.600-1.857)	0.850
**Presence**	**1.808 (1.022-3.202)**	**0.042**

Note: data after adjustment for sociodemographic factors including sex, age, smoking, education level, and exercise habit.

**Table 5:** The interaction effect between BS constitution and atherosclerotic factors on MMSE score.

Variables		*β*	R^2^	ΔR^2^	ΔF
Step 1			0.377	0.002	3.622^*∗*^
	RBC	0.126			
	HB	0.102			
	ABI	0.343^*∗∗*^			
	BaPWV	-0.053			
	BS constitution	-0.207^*∗*^			
Step 2			0.392	0.016	6.604^*∗∗∗*^
	RBC	0.165			
	HB	0.058			
	ABI	0.390^*∗∗∗*^			
	BaPWV	-0.066			
	BS constitution	-0.202			
	BS × RBC	-0.028			
	BS × HB	0.362^*∗*^			
	BS × ABI	0.348^*∗*^			
	BS × BaPWV	0.317^*∗*^			

Note: *∗*P<0.05, *∗∗*P<0.01, and *∗∗∗*P<0.001; *β* presents the unstandardized regression coefficient; R^2^ presents the fitting coefficient; ΔR^2^ and ΔF present the differences in R^2^ and F between two models. Data after adjustment for sociodemographic factors including sex, age, smoking, education level, and exercise habit.

## Data Availability

The data used to support the findings of this study are available from the corresponding author upon request.

## Supplementary Materials

Supplementary Materials Two short descriptions for appendixes are as follows. Appendix 1-MMSE-Form. The MMSE is a brief, quantitative measure of cognitive status in adults. It can be used to screen for cognitive impairment, to estimate the severity of cognitive impairment at a given point in time, to follow the course of cognitive changes in an individual over time, and to document an individual's response to treatment. Appendix 2-Constitution in TCM Questionnaire(33). The “English version Constitution in Traditional Chinese Medicine Questionnaire” is an effective research tool to carry out a large-scale research. It also can be used as a physical fitness assessment tool on foreigners.
