# Supplementary material for: The Interaction Effect between Blood Stasis Constitution and Atherosclerotic Factors on Cognitive Impairment in Elderly People
Source: Evid Based Complement Alternat Med. 2018 Nov 11;2018:8914090. doi: 10.1155/2018/8914090 (PMC6252209; doi:10.1155/2018/8914090)

MINI-MENTAL STATE EXAMINATION

Resident: ______________________________ Date: __________

Examiner's Name: ______________________________

Resident Score	Maximum Score	
		**ORIENTATION**
______	5	What is the (year), (season), (date), (day), (month)?
______	5	Where are we (country), (state), (county), (city), (clinic)?
		**REGISTRATION**
______	3	Name 3 objects allotting one second to say each one. Then ask the resident to name all 3 objects after you have said them. Give one point for each correct answer. Repeat them until he/ she hears all 3. Count trials and record number.<br>APPLE BOOK COAT Trials: __________
		**ATTENTION AND CALCULATION**
______	5	Begin with 100 and count back by 7 (stop after 5 answers) 93, 86, 79, 72, 65. Score one point for each correct answer. If the resident will not perform this task, ask the resident to spell "WORLD" backwards (DLROW). Record the resident's spelling: ______________________________
		**RECALL**
______	3	Ask the resident to repeat the objects above (see Registration). Give one point for each correct answer.
		**LANGUAGE**
______	2	Naming: Show a pencil, and a watch and ask the resident to name them.
______	1	Repetition: Repeat the following: "No ifs, ands or buts."
______	3	Three Stage Command: Follow the three-stage command. "Take paper in your right hand; fold it in half; and put it on the table."
______	1	Reading: Read and obey the following: "Close your eyes." Show the resident the item written on the reverse side, or attached.
______	1	Writing: Write a sentence (on reverse side)
______	1	Copying: Copy the design of the intersecting pentagons (on reverse side).
______	30	Total Score Possible

CLOSE YOUR EYES

WRITE A SENTENCE

COPY DESIGN

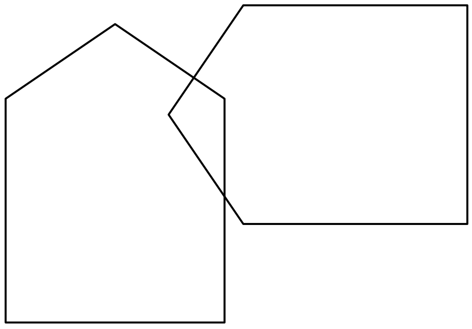

Supplement: Supplementary Materials — Two short descriptions for appendixes are as follows. Appendix 1-MMSE-Form. The MMSE is a brief, quantitative measure of cognitive status in adults. It can be used to screen for cognitive impairment, to estimate the severity of cognitive impairment at a given point in time, to follow the course of cognitive changes in an individual over time, and to document an individual's response to treatment. Appendix 2-Constitution in TCM Questionnaire(33). The “English version Constitution in Traditional Chinese Medicine Questionnaire” is an effective research tool to carry out a large-scale research. It also can be used as a physical fitness assessment tool on foreigners. [file 8914090.f1.zip › Appendix 1-MMSE-Form.pdf]
