# Supplementary material for: The Interaction Effect between Blood Stasis Constitution and Atherosclerotic Factors on Cognitive Impairment in Elderly People
Source: Evid Based Complement Alternat Med. 2018 Nov 11;2018:8914090. doi: 10.1155/2018/8914090 (PMC6252209; doi:10.1155/2018/8914090)
Supplement: Supplementary Materials — Two short descriptions for appendixes are as follows. Appendix 1-MMSE-Form. The MMSE is a brief, quantitative measure of cognitive status in adults. It can be used to screen for cognitive impairment, to estimate the severity of cognitive impairment at a given point in time, to follow the course of cognitive changes in an individual over time, and to document an individual's response to treatment. Appendix 2-Constitution in TCM Questionnaire(33). The “English version Constitution in Traditional Chinese Medicine Questionnaire” is an effective research tool to carry out a large-scale research. It also can be used as a physical fitness assessment tool on foreigners. [file 8914090.f1.zip › Appendix 2-Constitution in TCM Questionnaire(33).pdf]

中医体质辨识量表

Constitution in Traditional Chinese Medicine Questionnaire

问 题 / Items	1-没有/no	2-很少 /little	3-有时 /sometime	4-经常 /often	5-总是 /always
(1)您精力充沛吗？<br>Were you energetic?	1	2	3	4	5
(2)您容易疲乏吗？<br>Did you get tried easily?	1	2	3	4	5
(3)您容易气短，呼吸短促，接不上气吗？<br>Did you suffer from shortness of breath?	1	2	3	4	5
(4)您说话声音低弱无力吗?<br>Do you feel weak when talking?	1	2	3	4	5
(5)您感到闷闷不乐、情绪低沉吗?<br>Did you feel gloomy and depressed?	1	2	3	4	5
(6)您容易精神紧张、焦虑不安吗?<br>Do you get anxious and worried easily?	1	2	3	4	5
(7)您因为生活状态改变而感到孤独、失落吗？<br>Did you feel lonely, vulnerable or emotionally upset due to lifestyle changed?	1	2	3	4	5
(8)您容易感到害怕或受到惊吓吗?<br>Were you easily scared or frightened?	1	2	3	4	5
(9)您感到身体超重不轻松吗?<br>Did you feel heavy or lethargic?	1	2	3	4	5
(10)您眼睛干涩吗?<br>Did your eyes feel dry and use eye drops?	1	2	3	4	5
(11)您手脚发凉吗?<br>Did your body feel cold or clammy?	1	2	3	4	5
(12)您胃脘部、背部或腰膝部怕冷吗？<br>Did you feel cold easily in your abdomen, back, lower back or knees?	1	2	3	4	5
(13)您比一般人耐受不了寒冷吗？（指比别人容易害怕冬天或是夏天的冷空调、电扇等）<br>Did you feel more vulnerable to the cold than others (winter coldness, air conditioners, fans, etc) ?	1	2	3	4	5
(14)您容易患感冒吗?（指每年感冒的次数）<br>Did you catch colds more easily than others?	1<br>less than 2 times a year	2<br>2-4 times a year	3<br>5-6 times a year	4<br>more than 8 times a year	5<br>almost every month
(15)您没有感冒时也会鼻塞、流鼻涕吗?<br>Did you sneeze even when you did not have a cold?	1	2	3	4	5
(16)您有口粘口腻，或睡眠打鼾吗？<br>Did you sleep snoring or your mouth feel sticky?	1	2	3	4	5

(17)您容易过敏(对药物、食物、气味、花粉或在季节交替、气候变化时)吗?<br>Did you have allergies? (E.g. medicine, food, odors, pollen, pet dander, or during seasonal or weather change etc.)?	1<br>none	2<br>1-2 times a year	3<br>3-4 times a year	4<br>5-6 times a year	5<br>every time
(18)您的皮肤容易起荨麻疹吗?(包括风团、风疹块、风疙瘩)<br>Did your skin get hives/urticarial easily?	1	2	3	4	5
(19)您的皮肤在不知不觉中会出现青紫瘀斑、皮下出血吗?<br>Did black or purple bruises appear on your skin for no reason?	1	2	3	4	5
(20)您的皮肤一抓就红，并出现抓痕吗?<br>Did you skin turn red and show traces when you scratched it?	1	2	3	4	5
(21)您皮肤或口唇干吗?<br>Did your skin or lips feel dry?	1	2	3	4	5
(22)您有肢体麻木或固定部位疼痛的感觉吗？<br>Did you feel numbness in the limb or tingling pain somewhere in your body?	1	2	3	4	5
(23)您面部或鼻部有油腻感或者油亮发光吗?<br>Did your nose or face feel greasy, oily, or shiny?	1	2	3	4	5
(24)您面色或目眶晦黯，或出现褐色斑块/斑点吗?<br>Did you have dark face or get brown spots easily?	1	2	3	4	5
(25)您有皮肤湿疹、疮疖吗？<br>Did you get eczema or sores easily?	1	2	3	4	5
(26)您感到口干咽燥、总想喝水吗？<br>Did you often feel parched and need to drink water?	1	2	3	4	5
(27)您感到口苦或嘴里有异味吗?<br>Did you feel bitterness or a strange taste in your mouth?	1	2	3	4	5
(28)您腹部肥大吗?<br>Was you belly flabby?	1<br>waist circumference <80cm	2<br>waist circumference 80-85cm	3<br>waist circumference 86-90cm	4<br>waist circumference 91-105cm	5<br>waist circumference >105cm
(29)您吃(喝)凉的东西会感到不舒服或者怕吃(喝)凉的东西吗？<br>Did you easily contract diarrhea when you were exposed to cold or eat (or drink) something cold?	1	2	3	4	5
(30)您有大便黏滞不爽、解不尽的感觉吗?<br>Did you pass sticky stools and/or feel that your bowel movement is incomplete?	1	2	3	4	5

(31) 您容易便秘或大便干燥吗?<br>Did you get constipated easily or have dry stools?	1	2	3	4	5
(32) 您舌苔厚腻或有舌苔厚厚的感觉吗?<br>Did your tongue have a thick coating?	1	2	3	4	5
(33)您舌下静脉瘀紫或增粗吗？<br>Did you have dull purple or thickened hypoglossal vein?	1	2	3	4	5

**Unbalanced Constitution and Items**	**Conditions**	**Assessment results**
Qi-deficiency（02）（03）（04）（14）<br>Yang-deficiency（11）（12）（13）（29）<br>Yin-deficiency（10）（21）（26）（31）<br>Phlegm（09）（16）（28）（32）<br>Damp-heat（23）（25）（27）（30）<br>Blood-stasis（19）（22）（24）（33）<br>Qi-stagnation（05）（06）（07）（08）<br>Inherited Special（15）（17）（18）（20）	if the cumulative score of all items ≥11	Yes
	if the cumulative score of all items =9-10	Tendency
	if the cumulative score of all items ≤ 8	No
**Balanced Constitution**（01）（02）（04）（05）（13）<br>(Among them, (02) (04) (05) (13) reverse score, 1→5，2→4，3→3，4→2，5→1)	if the cumulative score of all items ⩾17 and the final score of each of the other eight constitutions < 8	Yes
	if the cumulative score of all items ⩾17 and the final score of each of the other eight constitutions < 10	Roughly yes
	Not meeting the above conditions	No
